# Tamoxifen attenuates dialysate-induced peritoneal fibrosis by inhibiting GSK-3β/β-catenin axis activation

**DOI:** 10.1042/BSR20180240

**Published:** 2018-11-21

**Authors:** Pengpeng Yan, Huanna Tang, Xiaoying Chen, Shuiyu Ji, Wei Jin, Jiaming Zhang, Jia Shen, Hao Deng, Xiang Zhao, Quanquan Shen, Hongfeng Huang

**Affiliations:** 1 Kidney Disease Center, The First Affiliated Hospital, College of Medicine, Zhejiang University, Hangzhou 310003, China; 2 Department of Nephrology, Zhejiang Provincial People’s Hospital, People’s Hospital of Hangzhou Medical College, Hangzhou 310014, China; 3 Department of Nephrology, Tongxiang First People’s Hospital, Jiaxing 314500, China

**Keywords:** epithelial-to-mesenchymal transition, β-catenin, GSK-3β, peritoneal fibrosis, tamoxifen

## Abstract

Peritoneal fibrosis is a severe complication arising from long-term peritoneal dialysis (PD). Tamoxifen (Tamo) has been clinically proven effective in a series of fibrotic diseases, such as PD-associated encapsulating peritoneal sclerosis (EPS), but the mechanisms underlying Tamoxifen’s protective effects are yet to be defined. In the present study, C57BL/6 mice received intraperitoneal injections of either saline, 4.25% high glucose (HG) PD fluid (PDF) or PDF plus Tamoxifen each day for 30 days. Tamoxifen attenuated thickening of the peritoneum, and reversed PDF-induced peritoneal expression of E-cadherin, Vimentin, matrix metalloproteinase 9 (MMP9), Snail, and β-catenin. Mouse peritoneal mesothelial cells (mPMCs) were cultured in 4.25% glucose or 4.25% glucose plus Tamoxifen for 48 h. Tamoxifen inhibited epithelial-to-mesenchymal transition (EMT) as well as phosphorylation of glycogen synthase kinase-3β (GSK-3β), nuclear β-catenin, and Snail induced by exposure to HG. TWS119 reversed the effects of Tamoxifen on β-catenin and Snail expression. In conclusion, Tamoxifen significantly attenuated EMT during peritoneal epithelial fibrosis, in part by inhibiting GSK-3β/β-catenin activation.

## Introduction

Peritoneal dialysis (PD) is a cost-effective renal replacement therapy for end-stage renal disease (ESRD) [1]. However, the peritoneum undergoes progressive injury from long-term exposure to bio-incompatible high-glucose PD fluid (PDF) leading to ultrafiltration failure and solute transport abnormalities associated with peritonitis and peritoneal fibrosis [2,3]. The pathological process involves myofibroblast generation from epithelial-to-mesenchymal transition (EMT) of peritoneal mesothelial cells (PMCs), contributing to increased rates of peritoneal transport and peritoneal fibrosis [4–6]. EMT is characterized by non-epithelial cell morphology and altered protein expression patterns including reduced levels of epithelial markers E-cadherin, cytokeratin, and zonula occludens-1 (ZO-1) and increased levels of mesenchymal markers N-cadherin, Vimentin, fibronectin, and Snail [6–9]. Long-term PD also promotes neoangiogenesis and vasculopathy [10], which further contribute to peritoneum dysfunction.

Tamoxifen is a selective estrogen receptor modulator initially used for the treatment of breast cancer [11]. It also displays anti-fibrotic activity in disorders such as retroperitoneal fibrosis [12], encapsulating peritoneal sclerosis (EPS) [13], sclerosing mesenteritis [14], sclerosing cervicitis, and fibrosing mediastinitis [15]. Tamoxifen’s anti-fibrosis activity has been linked to inhibition of contributing pathological processes including EMT and angiogenesis [6] and connective tissue growth factor promotion of collagen synthesis [16], and stimulation of metalloproteinase-9 degradation of collagen [17]. However, the molecular mechanisms underlying Tamoxifen’s effects remain unclear. β-catenin signaling is important during normal embryonic development and in homeostatic self-renewal [18] and has recently been implicated in the initiation of EMT [9] in renal fibrosis [19], pulmonary fibrosis [20], liver fibrosis [21], and skin fibrosis [22]. Conversely, glycogen synthase kinase-3β (GSK-3β) plays a key role in inhibiting β-catenin signaling activation in the above-mentioned pathologies [23]. It is not yet understood whether the anti-fibrotic effects of Tamoxifen in dialysis-induced peritoneal fibrosis are mediated through modulation of β-catenin signaling. In the present study, we examined the effects of Tamoxifen on EMT during high glucose (HG)-induced fibrosis in a C57BL/6 mouse model, on cultured mouse PMCs (mPMCs) exposed to HG and on GSK-3β/β-catenin axis stimulation.

## Materials and methods

### Animals and reagents

The experimental protocol was approved by the Ethics Committee for the Use of Experimental Animals in Zhejiang University (No. 2016-279) and was carried out in accordance with the National Institutes of Health Guide for the Care and Use of Laboratory Animals (NIH Publication No. 80-23). Eight-week-old male C57BL/6 mice (weight: 23 ± 2 g) were obtained from the Experimental Animal Center of Zhejiang Academy of Medical Sciences (Hangzhou, Zhejiang, China). Animals had free access to water and standard rodent chow and were maintained under a 12-h light/dark cycle [24].

The 4.25% HG PDF was purchased from Baxter (Guangzhou, Guangdong, China). Tamoxifen (Tamo) and TWS119 (TWS) were purchased from Selleckchem (Houston, Texas, U.S.A.). Antibodies against β-catenin (8480), p-β-catenin (8814), GSK-3β (12456), p-GSK-3β (9323), E-cadherin (14472), and β-tubulin (15115) were purchased from Cell Signaling Technology (Danvers, Massachusetts, U.S.A.). Antibodies against Vimentin (ab8978) and Snail (ab167609) were purchased from Abcam (Cambridge, Massachusetts, U.S.A.). Antibodies against fibronectin (15613), matrix metalloproteinase 9 (MMP9, 10375) and vascular endothelial growth factor (VEGF, 19003) for immunoblotting were purchased from Proteintech (Chicago, Illinois, U.S.A.). Antibodies against fibronectin (GB13091), MMP9 (GB13132), and cluster of differentiation 31 (CD31, GB11063) for histological staining were purchased from Servicebio (Wuhan, Hubei, China).

### PDF-induced peritoneal fibrosis in mice and Tamoxifen administration

Fifteen male C57BL/6 mice were randomly divided into three groups. Control mice received a daily intraperitoneal injection of saline (10% v/w, administered in lower right quadrant). Mice in the PDF and PDF + Tamo groups received equal volumes of PDF without or with Tamo (5 mg/kg/day), respectively. Peritoneal catheters were not implanted because they increased peritoneal fibrosis independent of PDF administration [24]. Following 30 days of treatment, mice were anesthetized with pentobarbital sodium and killed by cervical dislocation prior to harvesting peritoneum from the left half of the abdomen.

### Cell culture and treatments

mPMCs were collected from non-anesthetized male C57BL/6 mice by needle aspiration for 5 min following intraperitoneal injection of 2 ml trypsin-EDTA (0.05%, Thermo Fisher). Freshly aspirated mPMCs were grown in DMEM/F12 with 10% FBS (Thermo Fisher) in 5% CO_2_ at 37°C. Subsequently, mPMCs were incubated in serum-free medium for 12 h and then cultured with normal glucose (NG) or 4.25% d-glucose (HG) with or without Tamo (10 nM) for 48 h, followed by evaluation of E-cadherin, Vimentin, and GSK-3β expression. mPMCs were incubated for 12 h with NG, HG, or HG+Tamo in the absence or presence of TWS (1 μM) to inhibit GSK-3β activity, followed by evaluation of β-catenin, p-β-catenin, and Snail expression [25].

### Immunohistochemistry and immunofluorescence assay

Peritoneal tissue was fixed in formaldehyde, dehydrated in ethanol, clarified in xylene, embedded in paraffin, and sliced into 4-μm thick sections as described previously. The degree of peritoneal fibrosis was evaluated by Masson’s trichrome staining as described previously [26]. Photomicrographs (ten per animal) were taken using light microscopy (Leica) and analyzed using NIH ImageJ software. The thickness of peritoneum was calculated as the mean of five random measurements from the superficial mesothelial cell layer to the upper border of muscle.

Paraffin sections were blocked with 5% normal goat serum at room temperature for 1 h, incubated with primary antibodies against Vimentin, fibronectin, or MMP9 at 4°C overnight, washed in 1× PBS and incubated with the appropriate secondary antibody at room temperature for 30 min. Sections (ten per animal) were visualized and captured using light microscopy (Leica).

Frozen sections and mPMCs were fixed with 4% paraformaldehyde for 10 min at room temperature. After washing, the samples were blocked with 5% normal goat serum for 1 h and then incubated with primary antibodies against Vimentin, CD31, GSK-3β, Snail, or β-catenin overnight at 4°C. Subsequently, the appropriate fluorescently labeled secondary antibodies were added and incubated for 30 min at room temperature. Nuclei were stained with Hoechst (1:80000; Thermo Fisher). Images (ten per animal) were captured using a Leica fluorescence microscope.

### Western blotting

Mouse tissues and mPMCs were homogenized in RIPA lysis buffer with protease and phosphatase inhibitors (CST). Proteins were separated by 10% SDS/PAGE and electrotransferred to nitrocellulose membranes. The membranes were blocked with 5% non-fat milk and probed with primary antibodies against E-cadherin, Vimentin, MMP9, fibronectin, GSK-3β, p-GSK-3β, β-catenin, p-β-catenin, Snail, and β-tubulin (loading control), followed by appropriate labeled secondary antibodies. ECL reagent used for p-β-catenin was CST SignalFire™ Plus ECL Reagent (12630), for all other proteins CST SignalFire™ ECL Reagent (6883) was used. The membranes were scanned and band intensities were evaluated using Gel Doc XR (Bio-Rad).

### Statistical analysis

All experiments were repeated three times and presented as mean ± S.D. Standard ANOVA with Bonferroni’s post-test was used (GraphPad Prism 6.0). *P*<0.05 was considered statistically significant.

## Results

### EMT process was enhanced in PDF treated peritoneal membrane and HG treated mPMCs

**Figure 1:**
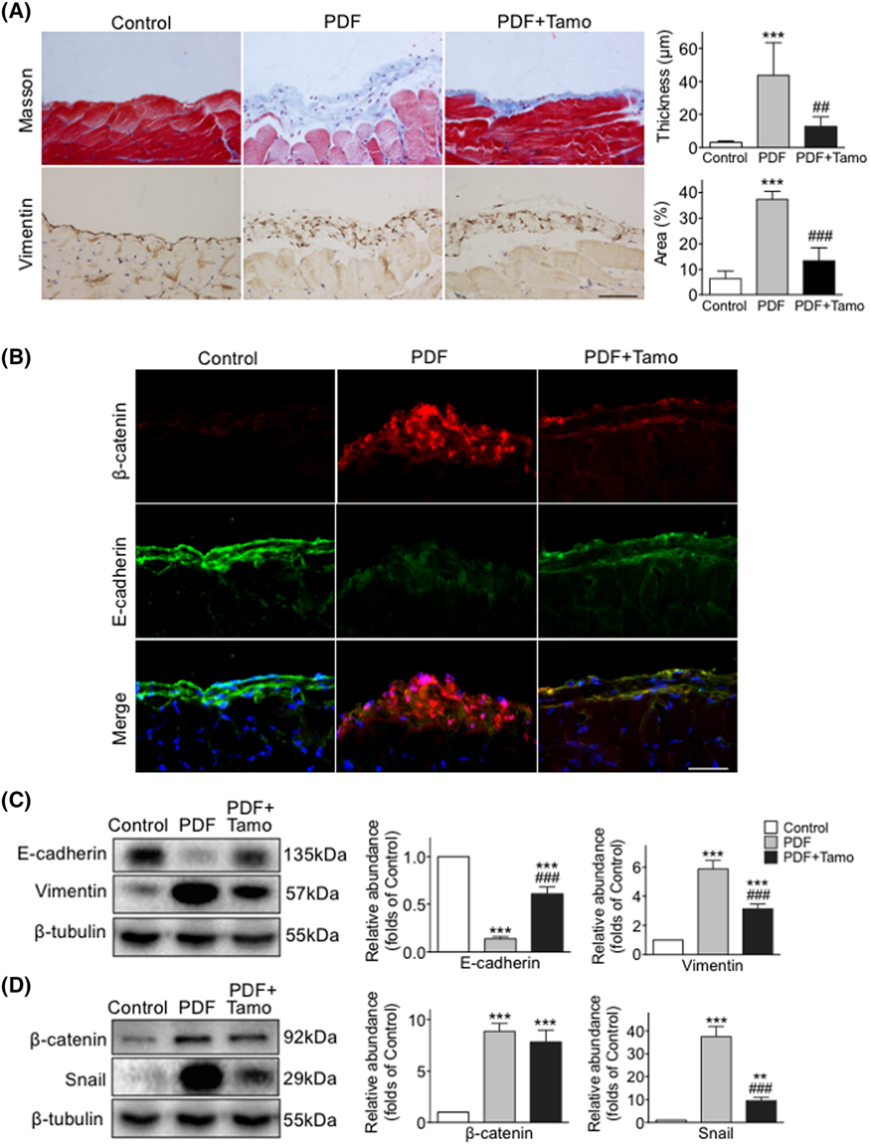
Tamoxifen attenuated EMT and decreased β-catenin expression in peritoneal fibrosis induced by HG PDF Peritoneal tissues collected after 30 days of exposure to 4.25% HG PDF with or without Tamoxifen (Tamo). (**A**) Paraffin sections stained with Masson’s trichrome (blue) or incubated with anti-Vimentin antibody (brown, left panel), and quantitated as the thickness and positive area of peritoneum (right panel). Original magnification: 400×. Bar = 100 μm. (**B**) Frozen sections were incubated with antibodies against β-catenin (red) and nuclei were stained with Hoechst (blue). Original magnification: 400×. Bar = 100 μm. (**C**,**D**) Expression levels of E-cadherin, Vimentin, β-catenin, and Snail examined by Western blotting (left panel) and quantitated by densitometry normalized to β-tubulin (right panel) (mean ± S.D., *n*=5, ***P<0.001 and **P<0.01 compared with control group, ^###^*P*<0.001 compared with PDF group).

**Figure 2:**
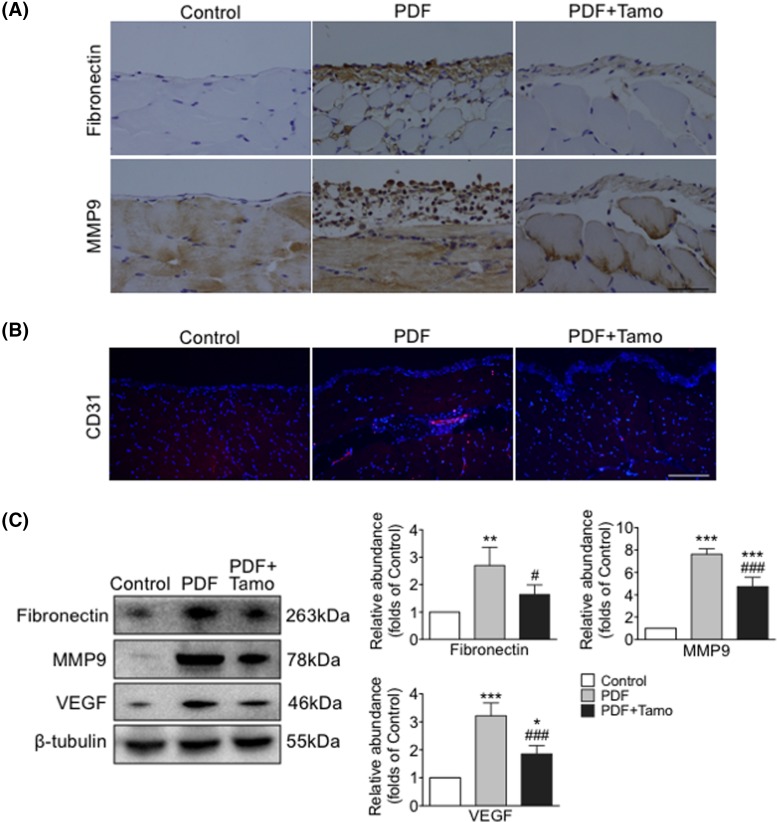
Tamoxifen suppressed up-regulation of fibronectin, MMP9, and angiogenesis following PDF exposure Peritoneal tissues collected after 30 days of exposure to 4.25% HG PDF with or without Tamoxifen (Tamo). (**A**) Paraffin sections incubated with anti-fibronectin or anti-MMP9 antibody (brown). Original magnification: 400×. Bar = 100 μm. (**B**) Frozen sections were incubated with antibodies against CD31 (red) and nuclei were stained with Hoechst (blue). Original magnification: 400×. Bar = 100 μm. (**C**) Expression levels of fibronectin, MMP9, and VEGF examined by Western blotting (left panel) and quantitated by densitometry normalized to β-tubulin (right panel) (mean ± S.D., *n*=5, ***P<0.001 and **P<0.01 compared with control group, ^###^*P*<0.001 compared with PDF group; *P<0.05 compared with control group and #*P*<0.05 compared with PDF group.).

**Figure 3:**
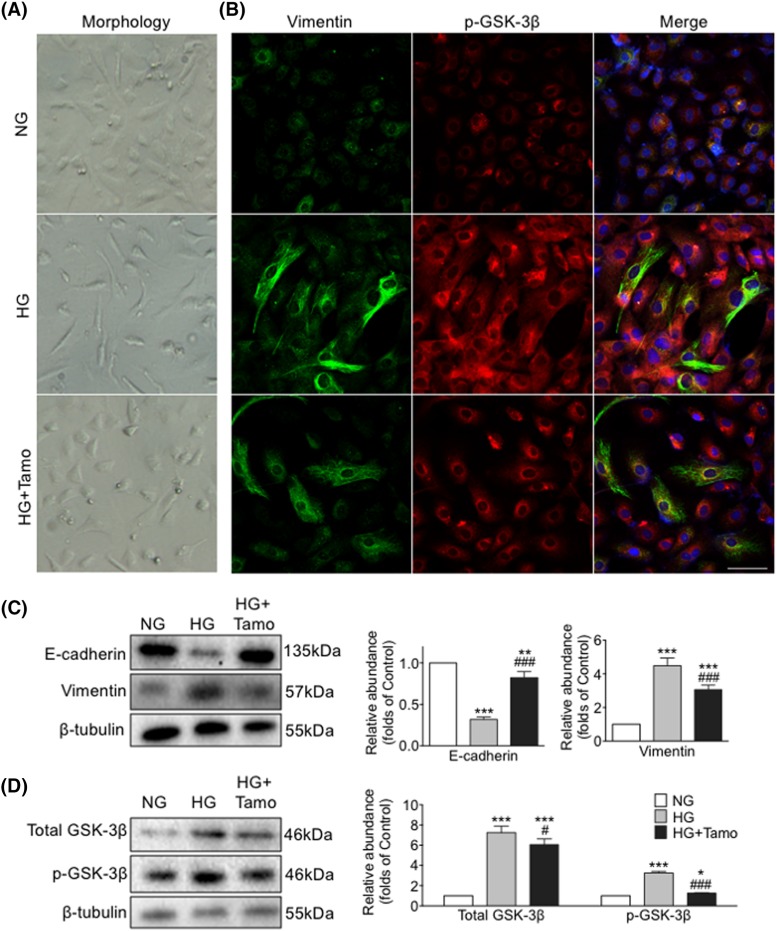
Tamoxifen reversed HG-induced mesenchymal morphology and EMT progression and restored GSK-3β activation in mPMCs mPMCs exposed to NG, HG, or combined HG plus Tamoxifen (HG + Tamo) for 48 h. (**A**) Phase-contrast microscopy showing cell morphology changes. (**B**) mPMCs incubated with antibodies against Vimentin (green) and p-GSK-3β (red). Nuclei were stained with Hoechst (blue). Bar = 100 μm. (**C**,**D**) Expression levels of E-cadherin, Vimentin, total GSK-3β, and p-GSK-3β examined by Western blotting (left panel) and quantitated by densitometry normalized to β-tubulin (right panel) (mean ± S.D., *n*=4, ***P<0.001, **P<0.01, and *P<0.05 compared with NG, ^###^*P*<0.001 and ^#^*P*<0.05 compared with HG).

In peritoneal tissue of PDF treated mice, levels of Vimentin, fibronectin, and MMP9 were increased while E-cadherin was reduced (Figures 1 and 2). In mPMCs, HG exposure led to increased Vimentin and β-catenin and reduced E-cadherin (Figure 3).

### Expression of β-catenin was up-regulated during EMT

**Figure 4:**
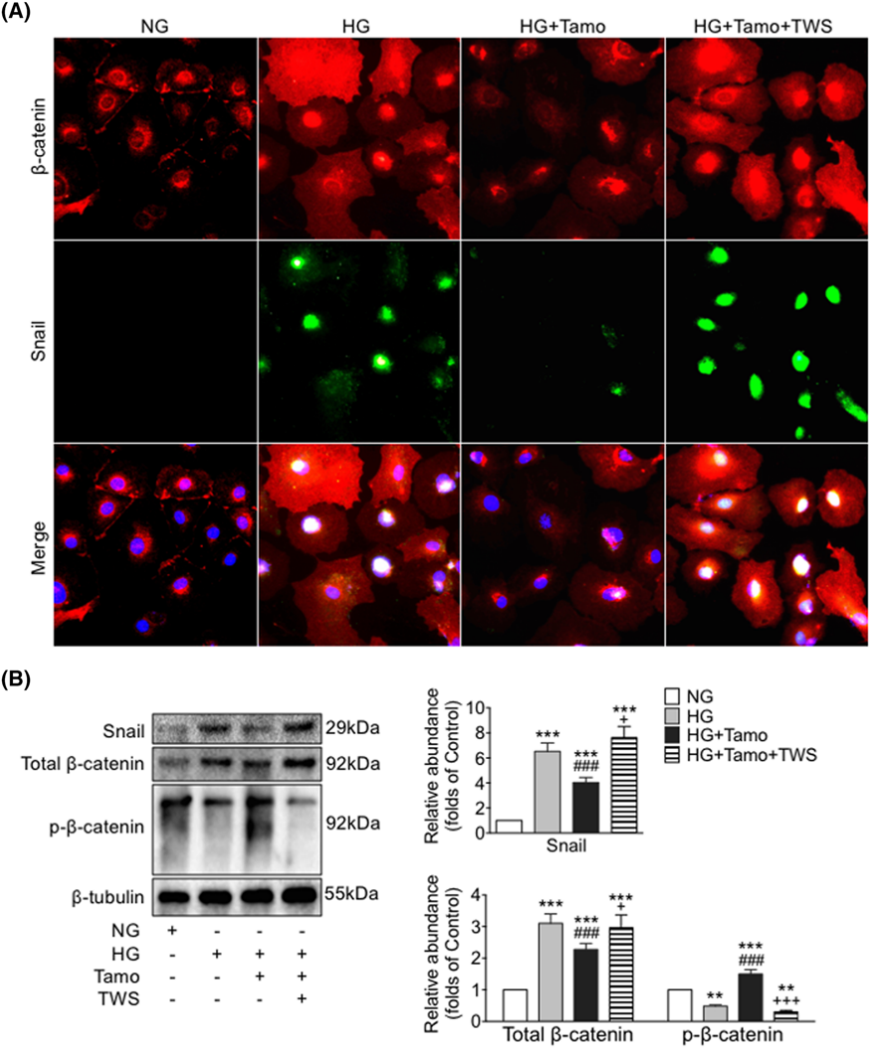
Tamoxifen inhibited HG-induced up-regulation and nuclear localization of β-catenin and Snail in mPMCs mPMCs exposed to NG, HG, HG plus Tamoxifen (HG + Tamo) and HG plus Tamoxifen plus TWS (HG + Tamo + TWS) for 12 h. (**A**) mPMCs incubated with antibodies against β-catenin (red) and Snail (green). Nuclei were stained with Hoechst (blue). Bar = 40 μm. (**B**) Expression levels of p-β-catenin, total β-catenin, and Snail in mPMCs examined by Western blotting (left panel) and quantitated by densitometry normalized to β-tubulin (right panel) (mean ± S.D., *n*=4, ***P<0.001 compared with NG, ^###^*P*<0.001 compared with HG, ^+^*P*<0.05 and ^+++^*P*<0.001 compared with HG + Tamo; **P<0.01 compared with NG.).

Protein expression of total β-catenin was increased in PDF treated peritoneal tissue and HG exposed mPMCs, while the level of inactive p-β-catenin was decreased (Figures 1 and 4). Thus *in vivo* PDF treatment and *in vitro* HG exposure induced similar effects upon levels of β-catenin and Vimentin compared with E-cadherin.

### Tamoxifen attenuated PDF-induced peritoneal fibrosis and peritoneal membrane EMT

An obvious thickening of peritoneal interstitium with collagen accumulation was observed following repeated injections of HG PDF, as indicated by Masson’s trichrome staining, and the degree of thickening was significantly lessened as a result of Tamoxifen treatment (Figure 1A). Co-treatment with Tamoxifen reversed the expression of EMT-associated proteins, including re-expression of E-cadherin and partial blockage of PDF-mediated up-regulation of Vimentin, Snail, fibronectin, and MMP9 (Figures 1 and 2). The number of CD31-positive cells and expression of VEGF were increased after PDF exposure, while the activity of new vessel formation was inhibited by Tamoxifen (Figure 2B,C).

### Tamoxifen inhibited HG-induced EMT in mPMCs

HG exposure induced morphological changes in mPMCs. Cells transitioned from a cobblestone-like morphology to a spindle shape, consistent with loss of epithelial characteristics and acquisition of a fibroblast-like phenotype during EMT. Treatment with Tamoxifen partially reversed this phenotypic transition (Figure 3A). Consistent with our *in vivo* observations, significantly increased Vimentin and decreased E-cadherin was observed in HG-treated mPMCs, and this change was attenuated by treatment with Tamoxifen (Figure 3B,C).

### Tamoxifen lowered phosphorylation of GSK-3β, nuclear translocation of β-catenin, and stimulation of Snail by HG

We sought to better understand the role of β-catenin-associated proteins in the development of peritoneal fibrosis induced by HG and as possible targets of Tamoxifen’s effects. Phosphorylation of GSK-3β was measured in mPMCs exposed to HG with and without Tamoxifen. We observed that levels of both total GSK-3β and p-GSK-3β were increased after HG exposure, and the increase was partially inhibited by Tamoxifen (Figure 3B,D). Moreover, significant overexpression of cytoplasmic and nuclear β-catenin and nuclear Snail was observed in mPMCs after HG exposure, which was markedly attenuated by Tamoxifen. Co-treatment with TWS, an inhibitor of GSK-3β, significantly reduced the effect of Tamoxifen. Levels of p-β-catenin, total β-catenin, and Snail were quantitated by Western blotting (Figure 4). HG induced expression of β-catenin target genes Snail, MMP9, and fibronectin were reduced after Tamoxifen administration (Figures 1D, 2, and 4).

## Discussion

In the present study, we observed that Tamoxifen treatment attenuated HG-induced EMT both *in vitro* and *in vivo*. In both PDF-treated peritoneum and HG-exposed mPMCs, Tamoxifen addition promoted maintenance of epithelial cell morphology and E-cadherin levels while reducing Vimentin expression. These results suggest that Tamoxifen partially attenuated fibrosis by inhibiting progression of EMT. Tamoxifen reduced phosphorylation of GSK-3β, activation and expression of β-catenin and Snail, and expression of β-catenin target genes fibronectin, Snail, and MMP9. The effects of Tamoxifen were themselves attenuated by the GSK-3β inhibitor TWS. These observations implicate the GSK-3β/β-catenin axis as a target of the anti-fibrotic effects of Tamoxifen.

β-catenin is maintained at low levels by a destruction complex comprising axin, adenomatous polyposis coli (APC), casein kinase 1a (CK1a), and GSK-3β. GSK-3β phosphorylation inhibits ubiquitylation activity of the destruction complex, resulting in accumulation of cytosolic β-catenin followed by migration into the nucleus [18,27,28]. Nuclear β-catenin forms a complex with T-cell factor/lymphoid enhancer binding factor (TCF/LEF) family members to stimulate transcription of target genes including *Snail, Twist, fibronectin, MMPs, Jagged1*, and *LEF1* [29,30], promoting cell proliferation, stem cell maintenance and differentiation [28]. It is well established that β-catenin is involved in EMT regulation during organ development and tumor metastasis [31–33] as well as wound healing and organ fibrosis [9,19]. MMP9 expression is closely correlated with submesothelial thickening and angiogenesis during peritoneum injury, possibly via E-cadherin processing leading to activation of β-catenin pathway signaling [34]. Snail is an important transcriptional regulator active during EMT associated with fibrosis, binding to the E-cadherin promoter and up-regulated as a result of β-catenin signaling [33]. Cytoplasmic Snail is stabilized by inhibition of GSK-3β-mediated phosphorylation [9], and nuclear Snail increases β-catenin target genes expression through a positive feedback loop [35]. Inhibition of either β-catenin or Snail blocked HG-induced EMT in tubular cells [36]. We previously showed that during EMT, phosphorylation of GSK-3β was enhanced in mPMCs by HG exposure accompanied by accumulation of cytoplasmic β-catenin. Nuclear co-localization of β-catenin and Snail was detected by immunofluorescence. These results suggest that the HG induced peritoneal fibrosis in C57BL/6 mice after long-term PDF injection, accompanied by phosphorylation of GSK-3β that increased expression, activation, and nuclear translocation of β-catenin and Snail and altered downstream EMT transcriptional factor levels. Thus, the β-catenin pathway can be implicated in promoting EMT during progression of HG-induced peritoneal fibrosis.

Tamoxifen is used to treat a variety of fibrosis disorders [12–15]. Multiple signaling pathways, including estrogen receptor α-mediated transforming growth factor (TGF)-β1/Smad and CTGF-related pathways, are affected by Tamoxifen’s anti-fibrotic activity [14,15,32]. We examined the therapeutic effects of Tamoxifen against peritoneal fibrosis and showed that Tamoxifen alleviated peritoneal membrane thickening and angiogenesis. Tamoxifen diminished the number of submesothelial new blood vessels, as indicated by CD31 staining, with a down-regulation of VEGF in mice peritoneum. In cultured mPMCs, HG-induced cell morphology changes from a paving stone/cobblestone shape into a fibroblast-like mesenchymal phenotype was partially reversed by Tamoxifen, and effects on E-cadherin and Vimentin levels suggested that Tamoxifen alleviated HG PDF-induced peritoneal fibrosis by reducing EMT [6]. Tamoxifen-associated dephosphorylation of GSK-3β, down-regulation and inactivation of β-catenin and Snail, and reversal of Tamoxifen’s effects by the GSK-3β inhibitor TWS together suggest that GSK-3β and downstream β-catenin and Snail activities are affected in Tamoxifen inhibition of EMT. However, the specific and direct targets of Tamoxifen’s anti-fibrotic effects remain undefined, requiring further investigations.

In conclusion, Tamoxifen significantly attenuated HG dialysate-induced peritoneal fibrosis via inhibition of EMT progression in peritoneum, at least in part through inhibition of GSK-3β/β-catenin axis activation.

## Abbreviations

CD31: cluster of differentiation 31
CST: cell signaling technology
CTGF: connective tissue growth factor
DMEM: Dulbecco’s modified Eagle mediau,
EMT: epithelial-to-mesenchymal transition
GSK-3β: glycogen synthase kinase-3β
HG: high glucose
MMP9: matrix metalloproteinase 9
mPMC: mouse peritoneal mesothelial cell
NG: normal glucose
RIPA: radio immunoprecipitation assay
PD: peritoneal dialysis
PDF: PD fluid
Tamo: Tamoxifen
TWS: TWS119
VEGF: vascular endothelial growth factor
